# First detection of porcine respirovirus 1 in Germany and the Netherlands

**DOI:** 10.1111/tbed.14100

**Published:** 2021-05-05

**Authors:** Leonard Schuele, Erley Lizarazo‐Forero, Hayley Cassidy, Katrin Strutzberg‐Minder, Jan Boehmer, Sabine Schuetze, Sandra Loebert, Claudia Lambrecht, Juergen Harlizius, Alex W. Friedrich, Silke Peter, John W. A. Rossen, Natacha Couto

**Affiliations:** ^1^ University of Groningen, University Medical Center Groningen Department of Medical Microbiology and Infection Prevention Groningen The Netherlands; ^2^ Institute of Medical Microbiology and Hygiene University of Tübingen Tübingen Germany; ^3^ IVD Innovative Veterinary Diagnostics (IVD GmbH) Seelze Germany; ^4^ Animal Health Services Chamber of Agriculture of North Rhine‐Westphalia Bad Sassendorf Germany; ^5^ Department of Pathology University of Utah School of Medicine Salt Lake City UT USA; ^6^ The Milner Centre for Evolution, Department of Biology and Biochemistry University of Bath Bath UK

**Keywords:** Europe, next‐generation sequencing, porcine parainfluenza virus 1, porcine respirovirus 1, shotgun metagenomics sequencing

## Abstract

Porcine respirovirus 1, also referred to as porcine parainfluenza virus 1 (PPIV‐1), was first detected in deceased pigs from Hong Kong in 2013. It has since then been found in the USA, Chile and most recently in Hungary. Information on the pathogenicity and global spread is sparse. However, it has been speculated to play a role in the porcine respiratory disease complex. To investigate the porcine virome, we screened 53 pig samples from 26 farms within the Dutch–German border region using shotgun metagenomics sequencing (SMg). After detecting PPIV‐1 in five farms through SMg, a real‐time reverse transcriptase PCR (RT‐qPCR) assay was designed, which not only confirmed the presence of the virus in 1 of the 5 farms but found an additional 6 positive farms. Phylogenetic analysis found the closest match to be the first detected PPIV‐1 strain in Hong Kong. The Dutch‐German region represents a significant area of pig farming within Europe and could provide important information on the characterization and circulation of porcine viruses, such as PPIV‐1. With its recent detection in Hungary, these findings suggest widespread circulation of PPIV‐1 in Central Europe, highlighting the need for further research on persistence, pathogenicity and transmission in Europe.

## 1 INTRODUCTION

Viruses that belong to the Paramyxoviridae family have been associated with respiratory symptoms in farm animals and may cross host species barriers (Welch et al., 2017). Porcine respirovirus 1, also referred to as porcine parainfluenza virus 1 (PPIV‐1), belongs to the Respirovirus genus within the Paramyxoviridae family. While the pathogenicity of PPIV‐1 in pigs is still unclear, it has been isolated from pigs with respiratory symptoms (Lau et al., 2013). Challenge trials that involved inoculating conventional and CD/CD piglets with PPIV‐1 revealed minimal morbidity and no mortality, despite considerable viral replication occurring in the infected animals (Welch et al., 2018). Yet, PPIV‐1 has been detected in co‐infections with swine influenza virus (SIV) and porcine reproductive and respiratory syndrome virus (PRRSV), which suggests it could also play a role within the porcine respiratory disease complex (Welch et al., 2017). Information on the global spread and epidemiology is sparse. Apart from China (Lau et al., 2013), PPIV‐1 was only detected in the United States of America (USA) in 2016 (Palinski et al., 2016), in Chile in 2020 (Agüero et al., 2020) and in Hungary also in 2020 (Dénes et al., 2020).

The Dutch‐German region represents a significant area of pig farming within Europe and could provide crucial information on the circulation of porcine viruses. The Food Pro‐tec‐ts project (Food protection technologies for trans‐boundary systems, work package TIC 2; (https://www.foodprotects.eu/projekt/arbeitspakete/tic2/) aims to improve the early warning strategies for infectious diseases. For this purpose, we evaluated the use of shotgun metagenomics (SMg) as a method for unbiased detection of pathogens, including viruses, in a collection of nasal swab (NS), blood serum (BS) and oral fluid (OF) samples from pigs within the Dutch‐German border region. After detecting PPIV‐1 in five farms through SMg, a real‐time reverse transcriptase PCR (RT‐qPCR) assay was designed and 13 other farms were tested.

## 2 MATERIALS AND METHODS

### 2.1 Sample collection and selection

Samples were collected between June 2017 and October 2018 from farms within the Dutch‐German border region under the Food Protects project. NS, OF and BS samples were collected from pigs in farms that monitored SIV, PRRSV and *Salmonella* spp. The NS and BS samples consisted of 5 pooled individual specimens that were obtained from pigs from the same age group and from the same herd. The OF samples were pen‐based (a rope was used to collect OF specimens from all animals within a pen). All samples were frozen at −20°C after collection, shipped on ice and then stored at −80°C.

### 2.2 Next‐generation sequencing

Samples were initially chosen for SMg based on positive RT‐qPCR results for SIV (VetMAX™​ Gold SIV Detection Kit, Life Technologies) and/or PRRSV (Virotype, Hilden, Qiagen), as they are clinically relevant porcine pathogens. Overall, 34 BS (from 10 farms), 4 NS (from 4 farms) and 15 OF samples (from 12 farms) were sequenced using SMg, resulting in a total of 53 samples from 26 farms. Nucleic acids were extracted using the QIAamp Viral RNA Mini Kit (Qiagen, Hilden, Germany), including in‐column DNase digestion. Complementary DNA was synthesized, as described previously (Kafetzopoulou et al., 2018). Short‐read sequencing (SRS) libraries were generated using the KAPA HyperPlus Kit (Roche, Basel, Switzerland). Additionally, viral enrichment with oligonucleotide bait probes was performed on a selected sample using the SeqCap ViroCap share developer panel (Wylie et al., 2015). SRS libraries were sequenced on a NextSeq 500, generating 76 base pair‐reads on a Mid Output Kit v2.5 (Illumina, San Diego, CA, USA). Furthermore, long‐read sequencing (LRS) libraries were generated using the Ligation Sequencing Kit (SQK‐LSK109) and sequenced on a MinION device on a FLO‐MIN106 R9.4.1 flow cell (Oxford Nanopore Technologies (ONT)). Lysis buffer served as a negative control.

To obtain rapid viral detection and taxonomic identification, raw reads were uploaded onto Taxonomer (IDbyDNA, San Carlos, CA, USA). Following the detection of PPIV‐1, sequenced reads were first trimmed, then mapped against a PPIV‐1 database derived from available complete or near‐complete genomes on GenBank (*n* = 10, 27/08/2020) using CLC Genomics Workbench v.20.0.4 (CLC) (Qiagen, Hilden, Germany). The resulting consensus sequences had to cover >300 nucleotides (nt) of the respective reference genome to be considered valid and were subsequently confirmed and characterized by NCBI BLAST. Assembly was only performed on the sample BS‐1 as it had a high abundance of PPIV‐1 reads, using the best PPIV‐1 hit as guidance on CLC.

### 2.3 RT‐qPCR assay for the detection of PPIV‐1

After detecting PPIV‐1 using SMg, RT‐qPCR primers were designed by aligning all the available PPIV‐1 sequences on GenBank (*n* = 21, 01/10/2020) using Geneious Prime software v2020.0.5 (Biomatters Ltd., Auckland, New Zealand). The following primers and probe were designed: forward 5′‐GCACCACCACCTCCTCTATT‐3′, reverse 3′‐GCCAAAATGGCAGGGTTRTT‐5′ and probe 5′‐FAM‐TGCTCTCACTCCTTTTAGAACTAAATGTG‐BHQ1*‐*3′. NC_025402 was used as a reference genome for the position of the primers in the nucleocapsid (N) gene, forward primer (114–133 nt), reverse primer (196–177 nt) and the probe (174–146 nt). The resulting target fragment was 83 nt covering the nucleotides 114 to 196 of the N gene. The RT‐qPCR was performed using the recommended settings for the Brilliant II RT‐qPCR Master Mix 1‐Step (Agilent Technologies, Texas, USA) with a MxPro30005P system (Agilent). Briefly, 400 nM of the forward and reverse primer and 200 nM of the probe were used to create a total reaction volume of 25 μL. The reaction was performed with the following cycling conditions: 50°C for 45 min, 95°C for 10 min, followed by 45 cycles of 95°C for 20 s, 55°C for 60 s and 72°C for 30 s. A cut‐off value of Ct ≤40 was applied. The RT‐qPCR was performed on samples that had sufficient sample material remaining following SMg. An additional 17 OF samples, either positive for SIV and/or PRRSV but not previously analysed with SMg, were also included. These 17 samples were collected from a further 13 different farms within the Dutch–German border region within the same time frame.

### 2.4 Phylogenetic analysis

To create the PPIV‐1 phylogenetic tree, the near‐complete assembled PPIV‐1 genome from this study and PPIV‐1 genomes from GenBank (*n* = 10) were aligned with MAFFT v7.313 (Katoh et al., 2002). Phylogenetic trees were inferred from the alignments with the maximum likelihood approach implemented in RAxML v8.2.10 (Stamatakis et al., 2014) under the general time reversible (GTR‐G) substitution model (Stamatakis et al., 2014) and bootstrap of 1,000 replicates. A phylogenetic analysis of the L (RdRp Polymerase) and the F (Fusion protein) genes was also performed.

## 3 RESULTS AND DISCUSSION

**FIGURE 1:**
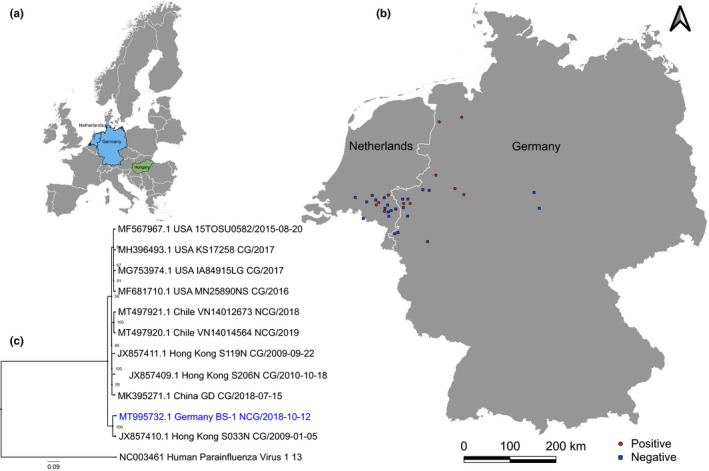
(a) European countries with reported PPIV‐1 detection. Hungary (green) in 2020, Germany and the Netherlands (blue) in 2020. (b) Relative geographic location of the pre‐selected German and Dutch farms. Farms with a PPIV‐1 detection are illustrated with a red square, and farms without a PPIV‐1 detection are illustrated with a blue hexagon. (c) Phylogenetic analysis of genome sequences of PPIV‐1. The phylogenetic tree was constructed using the sequence obtained in this study (blue) and all complete or near‐complete PPIV‐1 genomes available in GenBank (*n* = 10, 27/08/2020). Human parainfluenza virus 1 was used as an outgroup

The untargeted nature of SMg enables hypothesis‐free approaches for exploration, surveillance and characterization of the porcine virome. Using this approach, we detected PPIV‐1, an unexpected and rare virus, not previously seen in the Dutch–German border region, representing a major pig farming area in Europe (Figure 1a). Additionally, we designed a specific RT‐qPCR to test all available samples (both previously tested with and without SMg) to screen for PPIV‐1 in other farms.

Using the SMg approach, PPIV‐1 was detected in 5/53 samples (9.43%) from 3 different German farms and 2 different Dutch farms (5/26 farms; 19.2%) (Table 1). PPIV‐1 was not detected in the negative control. Using the RT‐qPCR approach, PPIV‐1 was detected in an additional 6 samples (OF3‐8) from two Dutch farms and four German farms from the SMg samples with sufficient sample left (*n* = 50), along with the extra 17 OF samples (Table 1). Positive farms are illustrated in Figure 1b. The PPIV‐1 RT‐qPCR resulted in Ct values varying from 27 to 37 (Table 1). One sample (OF‐2) was positive using the SMg and RT‐qPCR approaches. Sample BS‐1 did have a Ct value of 44, and however, this was above the RT‐qPCR cut‐off (Ct ≤40). The design of specific PPIV‐1 primers proved to be challenging due to the limited amount of available PPIV‐1 sequences on GenBank. Although the RT‐qPCR assay did reveal positive detection in samples not previously tested by SMg, it could not confirm PPIV‐1 detection in all SMg‐positive samples. A recently published paper designed a PPIV‐1 RT‐qPCR assay with primers based on the NCBI GenBank sequences, but contrary to our design, targeted the hemagglutinin–neuraminidase gene (Li et al., 2021). This RT‐qPCR approach should be able to detect the strains described in our study (in silico prediction). In the future, it could be applied to screen for PPIV‐1 and to pre‐select samples for NGS to obtain further complete or near‐complete sequences, not only to refine RT‐qPCR assays but also to ensure optimal surveillance. Overall, PPIV‐1 was detected in 1/34 BS samples (covering 10 farms), 2/4 NS samples (covering 4 farms) and 8/32 OF samples (covering 21 farms). In total, it was detected in 11 herds (31.4%) from 7 German farms and 4 Dutch farms by SMg and/or RT‐qPCR (Table 1).

**TABLE 1:** PPIV‐1‐positive sample overview

Sample ID	SMg PPIV−1 detection	RT‐qPCR Ct value			Country of origin	Date of sampling	Age (pen)	Symptoms (pen)
		PPIV−1^5^	SIV^6^	PRRSV^7^				
BS^1^−1	Yes	44*	NP^4^	26	Germany	10/2018	11 weeks	NA^8^
NS^2^−1	Yes	Neg	21	NP^4^	Germany	10/2018	Pig	NA^8^
NS^2^−2	Yes	Neg	19	NP^4^	Germany	10/2018	Pre‐fattening	Respiratory
OF^3^−1	Yes	NP^4^	36	30	Netherlands	08/2017	7 weeks	Respiratory
OF^3^−2	Yes	29	31	31	Netherlands	06/2017	Fattening pig	NA^8^
OF^3^−3	No	27	30	33	Netherlands	07/2017	9 weeks	NA^8^
OF^3^−4	No	35	34	29	Germany	08/2017	8 weeks	Respiratory
OF^3^−5	No	37	27	Neg	Germany	08/2017	Pig	NA^8^
OF^3^−6	NP^4^	29	25	Neg	Netherlands	08/2017	8 weeks	Respiratory
OF^3^−7	NP^4^	32	29	Neg	Germany	08/2017	NA^8^	Respiratory
OF^3^−8	NP^4^	29	27	34	Germany	08/2017	6 weeks	NA^8^

^1^ BS, blood serum (pooled).

^2^ NS, nasal swab (pooled).

^3^ OF, oral fluid (pen‐based)

^4^ NP, not performed.

^5^ PPIV‐1, porcine parainfluenza virus 1.

^6^ SIV, swine influenza virus.

^7^ PRRSV, porcine reproductive and respiratory syndrome virus.

^8^ NA, not available.

* Above cut‐off (Ct ≤40).

PPIV‐1 genome coverage obtained using SMg ranged from 5.5% to 99.7% (Table 2). The sequencing of host, environmental and non‐pathogenic nucleic acids, along with sequences of interest, reduces the sensitivity of SMg (Greninger, 2018). In this study, SMg did not detect viruses with Ct above 30. Therefore, viral enrichment strategies to obtain near‐ or complete viral sequences have been applied previously, including cell culture (Lau et al., 2013; Palinski et al., 2016) followed by depletion of ribosomal background RNA (Agüero et al., 2020). Application of oligonucleotide capture probes for viral enrichment on sample BS‐1, which had the highest number of reads, resulted in a 22.8‐fold increase in PPIV‐1 reads and enabled the assembly of a near‐complete genome sequence (15,344 nt) (GenBank accession: MT995732). This corresponded to 99.7% of the genome with a sequencing depth of 9,793 times. Furthermore, within BS‐1 we were also able to identify a co‐infection with PRRSV. Co‐infections with PRRSV and/or SIV have been reported previously (Lau et al., 2013; Welch et al., 2017), with PPIV‐1 being speculated to play a synergistic role within the porcine respiratory disease complex. However, the role of these co‐infections in the porcine respiratory disease complex remains to be ascertained (Park et al., 2019; Welch et al., 2017).

**TABLE 2:** Description of PPIV‐1‐positive samples using SMg.

Sample ID	NGS Platform	Best BLAST reference (length)	Identity (%)	Genome coverage (%)	Average sequencing depth
BS^1^−1	NextSeq	S033N (15,396 nt)	96.0	99.7	9,793
NS^2^−1	MinION	S033N (15,396 nt)	95.1	7.6	2
NS^2^−2	NextSeq	Gd2018 (15,396 nt)	97.0	2.4	1
OF^3^−1	MinION	S033N (15,396 nt)	96.2	33.0	3
OF^3^−2	NextSeq	S033N (15,396 nt)	89.5	5.5	1

^1^ BS, blood serum.

^2^ NS, nasal swab.

^3^ OF, oral fluid.

NCBI BLASTn analysis revealed that 4 out of the 5 PPIV‐1 sequences obtained from SMg had the highest similarity (89.5%–96.2%) to the Chinese PPIV‐1 strain S033N (GenBank accession: JX857410.1) (Table 2), which was first characterized from deceased pigs in Hong Kong in 2013 (Lau et al., 2013). Phylogenetic analysis of BS‐1 (MT995732) with other available complete or near‐complete genomes revealed clustering with strain S033N (Figure 1c). An additional phylogenetic analysis of partial F and L sequences from BS‐1, along with the 3 available PPIV‐1 strains from Hungary (Dénes et al., 2020), also showed the closest similarity to strain S033N (Figure S1). This could indicate that strains genetically related to S033N are established within Central Europe. In comparison, sample NS‐2 from a German farm showed the highest similarity (97.0%) to another Chinese PPIV‐1 strain, Gd2018 (GenBank accession: MK395271.1), which was collected in 2018.

Previously detected PPIV‐1 sequences were obtained from OF, NS and lung samples, with the upper respiratory tract being suggested to be the most suitable sampling site for detection (Agüero et al., 2020; Lau et al., 2013; Park et al., 2019). To the best of our knowledge, we have reported the first detection of PPIV‐1 in a BS sample. Interestingly, among the samples analysed with SMg, PPIV‐1 had the highest sequence read count in the BS sample, indicating an additional potential sampling matrix. Nevertheless, PPIV‐1 was only detected within 1/32 BS, compared to 2/4 NS and 8/32 OF samples. Additionally, in a study performed by Li and colleagues, RT‐qPCR analysis of 49 BS samples of PPIV‐1 infected pigs yielded no positive BS results (Li et al., 2021). As a result, it would suggest a limited suitability for detecting PPIV‐1 in BS, compared to NS and OF samples.

The aim of this study within the Food Protects project was to develop early warning methods for detection of swine pathogens. We chose SMg as the method to evaluate. The study design implied two limitations: firstly, farms had to be pre‐selected based on the suitability for long‐term monitoring and samples were further pre‐selected based on positive RT‐qPCR results for two clinically relevant pathogens, PRRSV and SIV. This pre‐selection bias prevents the breakdown of epidemiological links of PPIV‐1. Secondly, samples were either pooled (BS and NS) or pen‐based (OF) prior to testing. This is a trade‐off between an efficient method to monitor circulating pathogens on a herd level, however, at the same time, pathogens are unable to be linked to an individual animal within a herd.

In conclusion, to the best of our knowledge, we report the first detection of PPIV‐1 in Germany and the Netherlands, as well as the first near‐complete genome in Europe. Moreover, this is the first detection of PPIV‐1 using a targeted and untargeted NGS approach directly from the sample. As the PPIV‐1 sequences from Hungary, Germany and the Netherlands were closely related to strains previously found in China, it suggests there may have been PPIV‐1 transmission between Europe and China. Furthermore, as PPIV‐1 was detected in pigs from 11 different farms (5 using SMg and 6 using RT‐qPCR), it could confirm its circulation in Central Europe. Additional research is required to determine the extent of dissemination of PPIV‐1 in Europe, to assess its relevance in the porcine respiratory disease complex and its ability to cross host species barriers.

## CONFLICT OF INTEREST

John W. A. Rossen is employed by IDbyDNA. Silke Peter consults for IDbyDNA. This did not influence the interpretation of reviewed data and conclusions drawn nor on the drafting of the manuscript, and no support was obtained from them. All other authors declare no conflict of interest.

## ETHICAL APPROVAL

The animal samples used for this study were collected within the Food Pro‐tec‐ts project, classified as an animal study and approved on 22.09.2017 by the respective state office for nature, environment and consumer protection (file reference: 84.02.05.40.17.079).

## Supporting information

Supplementary Material

## Data Availability

The near‐complete PPIV‐1 sequence has been deposited under the GenBank accession: MT995732.

## References

[tbed14100-bib-0001] Agüero, B. , Mena, J. , Berrios, F. , Tapia, R. , Salinas, C. , Dutta, J. , … Neira, V. (2020). First report of porcine respirovirus 1 in South America. Veterinary Microbiology, 246, 108726. 10.1016/j.vetmic.2020.108726 32605754 PMC10898806

[tbed14100-bib-0002] Dénes, L. , Cságola, A. , Schönhardt, K. , Halas, M. , Solymosi, N. , & Balka, G. (2020). First report of porcine parainfluenza virus 1 (species Porcine respirovirus 1) in Europe. Transboundary and Emerging Diseases, 1–5. 10.1111/tbed.13869 33006252

[tbed14100-bib-0003] Greninger, A. L. (2018). The challenge of diagnostic metagenomics. Expert Review of Molecular Diagnostics, 18(7), 605–615. 10.1080/14737159.2018.1487292 29898605

[tbed14100-bib-0004] Kafetzopoulou, L. E. , Efthymiadis, K. , Lewandowski, K. , Crook, A. , Carter, D. , Osborne, J. , … Pullan, S. T. (2018). Assessment of metagenomic Nanopore and Illumina sequencing for recovering whole genome sequences of chikungunya and dengue viruses directly from clinical samples. Eurosurveillance, 23(50), 1800228. 10.2807/1560-7917.ES.2018.23.50.1800228 PMC6299504 30563591

[tbed14100-bib-0005] Katoh, K. , Misawa, K. , Kuma, K. , & Miyata, T. (2002). MAFFT: A novel method for rapid multiple sequence alignment based on fast Fourier transform. Nucleic Acids Research, 30(14), 3059–3066. 10.1093/nar/gkf436 12136088 PMC135756

[tbed14100-bib-0006] Lau, S. K. P. , Woo, P. C. Y. , Wu, Y. , Wong, A. Y. P. , Wong, B. H. L. , Lau, C. C. Y. , … Yuen, K. Y. (2013). Identification and characterization of a novel paramyxovirus, porcine parainfluenzavirus 1, from deceased pigs. Journal of General Virology, 94, 2184–2190. 10.1099/vir.0.052985-0 23918408

[tbed14100-bib-0007] Li, Y. , Sthal, C. , Bai, J. , Liu, X. , Anderson, G. , & Fang, Y. (2021). Development of a real‐time RT‐qPCR assay for the detection of porcine respirovirus 1. Journal of Virological Methods, 289, 114040. 10.1016/j.jviromet.2020.114040 33309757

[tbed14100-bib-0008] Palinski, R. M. , Chen, Z. , Henningson, J. N. , Lang, Y. , Rowland, R. R. R. , Fang, Y. , … Hause, B. M. (2016). Widespread detection and characterization of porcine parainfluenza virus 1 in pigs in the USA. Journal of General Virology, 97, 281–286. 10.1099/jgv.0.000343 26581410

[tbed14100-bib-0009] Park, J. Y. , Welch, M. W. , Harmon, K. M. , Zhang, J. , Piñeyro, P. E. , Li, G. , Hause, B. M. , & Gauger, P. C. (2019). Detection, isolation, and *in vitro* characterization of porcine parainfluenza virus type 1 isolated from respiratory diagnostic specimens in swine. Veterinary Microbiology, 228, 219–225. 10.1016/j.vetmic.2018.12.002 30593371

[tbed14100-bib-0010] Stamatakis, A. (2014). RAxML version 8: A tool for phylogenetic analysis and post‐analysis of large phylogenies. Bioinformatics, 30, 1312–1313. 10.1093/bioinformatics/btu033 24451623 PMC3998144

[tbed14100-bib-0011] Welch, M. , Park, J. , Gauger, P. , Harmon, K. , Lin, K. , Pineyro, P. , & Zhang, J. (2017). Porcine Parainfluenza Virus Type 1 (PPIV‐1) in US Swine: Summary of Veterinary Diagnostic Laboratory Data. Iowa State University Animal Industry Report, 14(1).

[tbed14100-bib-0012] Welch, M. , Harmon, K. M. , Zhang, H. L. , Piñeyro, P. , Gimenez‐Lirola, L. , & Gauger, P. (2018). Experimental efficacy of a Merck RNA particle (RP) vaccine against porcine parainfluenza type 1 (PPIV‐1) challenge in weaned piglets. In 49th American Association of Swine Veterinarians Annual Meeting (pp. 280–281). San Diego.

[tbed14100-bib-0013] Wylie, T. N. , Wylie, K. M. , Herter, B. N. , & Storch, G. A. (2015). Enhanced virome sequencing using targeted sequence capture. Genome Research, 25(12), 1910–1920. 10.1101/gr.191049.115 26395152 PMC4665012

