# Supplementary material for: First detection of porcine respirovirus 1 in Germany and the Netherlands
Source: Transbound Emerg Dis. 2021 May 5;68(6):3120–5. doi: 10.1111/tbed.14100 (PMC9292642; doi:10.1111/tbed.14100)
Supplement: Supplementary file 1 — Supplementary Material [file TBED-68-3120-s001.docx]

**
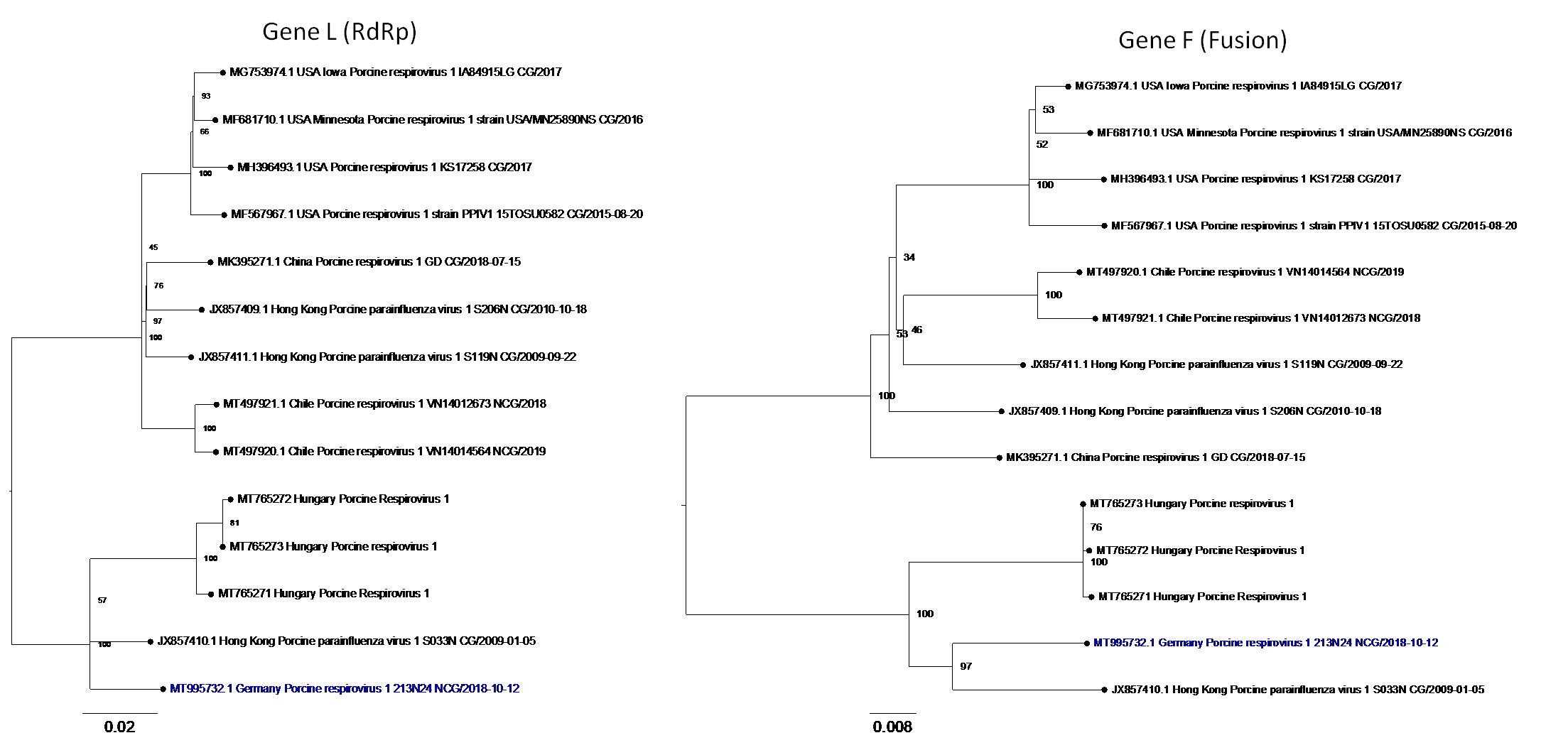
**

**FIGURE S1** Phylogenetic analysis of the PPIV-1 RNA polymerase (L) and fusion protein (F) genes. The phylogenetic tree was constructed using the sequences from the L and the F genes obtained in this study (blue) and all available in GenBank (n=13, 27/08/2020). The Hungarian strains (GenBank accession numbers: MT765271.1, MT765272.1, MT765273.1) are partial CDS sequences.
